# Delivering Beneficial Microorganisms for Corals: Rotifers as Carriers of Probiotic Bacteria

**DOI:** 10.3389/fmicb.2020.608506

**Published:** 2020-12-15

**Authors:** Juliana M. Assis, Fernanda Abreu, Helena M. D. Villela, Adam Barno, Rafael F. Valle, Rayssa Vieira, Igor Taveira, Gustavo Duarte, David G. Bourne, Lone Høj, Raquel S. Peixoto

**Affiliations:** ^1^Laboratory of Molecular Microbial Ecology, Institute of Microbiology Paulo de Góes, Universidade Federal do Rio de Janeiro, Rio de Janeiro, Brazil; ^2^Laboratory of Cellular Biology and Magnetotaxis, Institute of Microbiology Paulo de Góes, Universidade Federal do Rio de Janeiro, Rio de Janeiro, Brazil; ^3^IMAM-AquaRio – Rio de Janeiro Aquarium Research Center, Rio de Janeiro, Brazil; ^4^Australian Institute of Marine Science, Townsville, WA, Australia; ^5^College of Science and Engineering, James Cook University, Townsville, QLD, Australia

**Keywords:** Beneficial Microorganisms for Corals (BMCs), rotifers, marine probiotics, *Brachionus plicatilis*, *Pocillopora damicornis*, delivery, coral reefs, microscopy

## Abstract

The use of Beneficial Microorganisms for Corals (BMCs) to increase the resistance of corals to environmental stress has proven to be effective in laboratory trials. Because direct inoculation of BMCs in larger tanks or in the field can be challenging, a delivery mechanism is needed for efficient transmission of the BMC consortium. Packaged delivery mechanisms have been successfully used to transmit probiotics to other organisms, including humans, lobsters, and fish. Here, we tested a method for utilizing rotifers of the species *Brachionus plicatilis* for delivery of BMCs to corals of the species *Pocillopora damicornis*. Epifluorescence microscopy combined with a live/dead cell staining assay was used to evaluate the viability of the BMCs and monitor their *in vivo* uptake by the rotifers. The rotifers efficiently ingested BMCs, which accumulated in the digestive system and on the body surface after 10 min of interaction. Scanning electron microscopy confirmed the adherence of BMCs to the rotifer surfaces. BMC-enriched rotifers were actively ingested by *P. damicornis* corals, indicating that this is a promising technique for administering coral probiotics *in situ*. Studies to track the delivery of probiotics through carriers such as *B. plicatilis*, and the provision or establishment of beneficial traits in corals are the next proof-of-concept research priorities.

## Introduction

Coral reefs are increasingly impacted by global climate change, which raises the mean sea surface temperature (SST) and the incidence of marine heatwaves (Hughes et al., 2018). Due to increases in the duration and intensity of these thermal stress events, higher rates of coral mortality have been reported globally (Pandolfi et al., 2011; Duarte et al., 2020). Efforts to mitigate the negative effects of global changes on coral reefs have led to the implementation of different strategies in conservation studies. A report published by the United States National Academies of Sciences, Engineering, and Medicine listed several methods of intervention that are currently being studied and developed to increase coral resistance and resilience (National Academies of Sciences and Medicine, 2019). One promising approach is the manipulation of different coral-associated microbes to increase host resistance and resilience to stressors (Peixoto et al., 2017; National Academies of Sciences and Medicine, 2019). This strategy relies on key host–microbiome symbiotic relationships that can be exploited to increase the fitness of the coral holobiont (Peixoto et al., 2017; Pita et al., 2018; Wilkins et al., 2019). Coral-associated bacteria have been shown to fix nitrogen, degrade polysaccharides, and produce antimicrobial compounds that can inhibit pathogen growth (reviewed by Peixoto et al., 2017). Although the ecological relationships, taxonomic composition, and metabolic pathways of microbial communities associated with corals have been determined (Rohwer et al., 2001; Bourne and Munn, 2005; Sánchez-Quinto and Falcón, 2019), the selection and use of specific microorganisms as probiotics for corals on an ecologically relevant scale is a relatively new field of research (Teplitski and Ritchie, 2009; Santos et al., 2015; Peixoto et al., 2017; Damjanovic et al., 2019; Rosado et al., 2019).

Selection and manipulation of specific members of the resident coral microbiome to mitigate the effects ofthermal stress on the animal health was proposed by Peixoto et al. (2017). The coral-associated bacteria are chosen as beneficial microorganisms involved in the protection, health maintenance, and growth of corals (Rosado et al., 2019). Administering Beneficial Microorganisms for Corals (BMCs) has helped to increase coral resistance against different threats, such as oil contamination, disease, and thermal stress (Santos et al., 2015; Rosado et al., 2019). In these proof-of-concept experiments, a consortium containing BMCs was concentrated and applied directly to corals and the surrounding water, without a biological carrier system (Rosado et al., 2019). Although possible delivery systems have been proposed for closed- and open-water systems (see Peixoto et al., 2017), there is still a lack of studies on potential effective strategies for BMC delivery to corals in aquarium or field settings.

**FIGURE 1:**
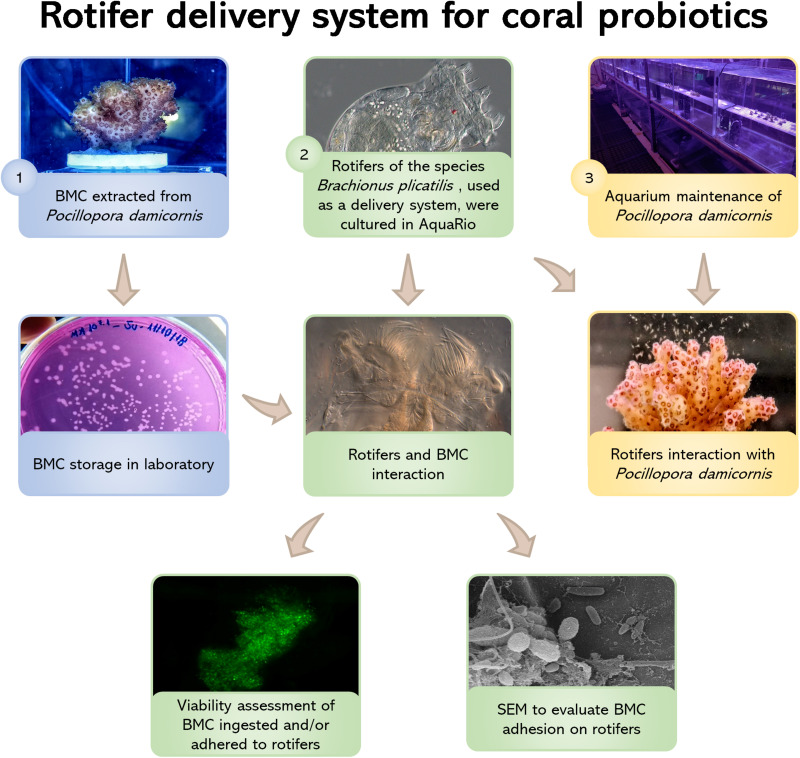
Flowchart showing all treatment steps: (1) BMC strains were isolated from the coral *Pocillopora damicornis* by Rosado et al. (2019) and deposited in the microbial collection of the Microbial Molecular Ecology Laboratory (MMEL), UFRJ, Rio de Janeiro, Brazil; (2) Rotifers *Brachionus plicatilis* were cultured at the Rio de Janeiro Marine Aquarium Research Center (AquaRio) and incubated with the BMCs from the MMEL collection. Two different methods were used to assess rotifer-BMC interactions: fluorescence microscopy (using the LIVE/DEAD *Bac*Light^TM^ Bacterial Viability Kit) and scanning electron microscopy (SEM); (3) Nubbins of *P. damicornis* were cultured at AquaRio and transferred to aquarium for the interaction experiment with rotifers *B. plicatilis*. Video and still images of the polyps capturing the rotifers show that the corals were able to take up the rotifers.

In humans, probiotic foods and beverages are common ways to deliver *Lactobacillus* species (Roobab et al., 2020) and other beneficial bacteria to improve health (Dunne et al., 1999; Casas and Dobrogosz, 2000; Stolzenbach et al., 2020). Likewise, small organisms such as brine shrimp (*Artemia*), rotifers, and copepods can be used as vectors for transmitting probiotic bacteria to larger animals in aquaculture, such as fish (Planas et al., 2005, 2006; Sun et al., 2013; Hai, 2015), prawns (Hai et al., 2010), and lobsters (Daniels et al., 2013). Here, we evaluated the potential of the rotifer *Brachionus plicatilis* (Figure 1 and Supplementary Figure 1) as a vector for delivering BMCs into corals by feeding the rotifers with a previously validated BMC consortium assembled by Rosado et al. (2019), and tracking the uptake of rotifers by the coral *Pocillopora damicornis*.

## Materials and Methods

The steps described in the next topics are summarized in the flowchart shown in Figure 1.

### Rotifer and Coral Cultures

*Brachionus plicatilis* rotifers and *P. damicornis* nubbins were cultured at the Rio de Janeiro Marine Aquarium Research Center (AquaRio). Rotifers were cultured in natural seawater, at a temperature of 25°C, salinity of 3.2%, in the dark. Water circulation and oxygenation were produced by bubbling air with an air pump. Using standard culture conditions, rotifers were fed daily with 100 mL of a culture of the alga *Tetraselmis gracilis* containing 10^6^ cells mL^–1^. Conversely, before starting the experiment, rotifers were transferred to 1 L containers with oxygenation produced by bubbling air with an air pump. The container was kept in the dark to avoid the growth of photosynthetic organisms. Rotifers were kept under starvation by culturing them in 0.22-μm daily filtered seawater, for a 7-day period.

Corals were kept in tanks containing 100L of seawater at 25°C, salinity of 3.2%, light intensity of 200 μmol m^–2^ s^–1^, and water recirculation by circulation pumps. Twenty percent of the water in the coral tank system was exchanged every 7 days. Corals were fed daily with 100 mL of a culture containing 200 rotifers mL^–1^ under standard culture conditions (i.e., not starved).

### BMC Uptake by Rotifers

The seven strains of bacteria used in the experiment were isolated by Rosado et al. (2019) and stored in the microbial collection of the Microbial Molecular Ecology Lab (MMEL), UFRJ, Rio de Janeiro, Brazil. This consortium was composed of five strains of *Pseudoalteromonas* spp., isolated from *P. damicornis*; plus one strain of *Cobetia marina* and one strain of *Halomonas taeanensis*, isolated from the artificial seawater surrounding *P. damicornis*. The accession numbers of each isolate and their classifications according to their 16S rRNA gene sequences are provided in Supplementary Table 1.

### Localization of the BMC Consortium Associated With Rotifers

To prepare the BMC consortium assembled by Rosado et al. (2019), individual BMC strains were first grown in Marine Agar (MA) medium to evaluate purity. Sterile inoculation loops were used to pick single bacterial colonies of each strain from the MA plates, and the cells were inoculated into 5 mL of Marine Broth (MB) medium. Cultures were grown overnight at 26°C, and 1% (v/v) of each strain was individually inoculated into 100 mL of MB medium in a 250-mL culture flask and incubated at 26°C with agitation of 100 rpm until each culture reached 10^7^ cells mL^–1^. The cells were collected by centrifugation at 12,000 × *g* for 5 min at 4°C and washed twice under the same conditions with sterile saline (2.5% w/v NaCl) to remove the culture medium. All cells from each culture were homogenized together, resuspended in a final volume of 40 mL of saline solution (2.5% w/v NaCl), and stored at 4°C until used in the experiment, which occurred on the same day of the preparation of the cells.

Fluorescence and differential interference contrast (DIC) images were obtained with a Zeiss Axio Imager D2 microscope (Zeiss, Germany). Fluorescence filters used were: (i) Filter set 10 (excitation: BP 332 450–490 nm; beam splitter: FT 510 nm; emission: LP 515 nm); and (ii) Filter set 00 (excitation: BP 546/12; beam splitter: FT 560; emission: BP 575–640 nm).

Based on initial trials, rotifers were starved for 7 days before use in experiments, to limit their autofluorescence by food ingestion. The viability of the rotifers after 7 days of starvation was determined by observing their mobility and cilia beating, using DIC microscopy. The LIVE/DEAD^TM^ Bacterial Viability Kit (Thermo-Fisher Scientific, United States) was used to stain BMCs, following the manufacturer’s protocol. BMCs were washed in sterile seawater to remove excess stain prior to delivery to the rotifers. For BMC-rotifer interactions, the stained BMC homogenized consortium (100 μL of a suspension containing approximately 10^7^cells) was inoculated into a 2-mL polypropylene tube containing 900 μL of seawater and a concentration of 70 rotifers mL^–1^. Promptly after inoculation (around 10 min), a 20-μL aliquot of BMC-rotifer suspension was mounted on a glass slide, alongside a control with unstained bacteria, and both preparations were observed by means of fluorescence microscopy.

The interactions between rotifers and BMCs were also observed using scanning electron microscopy (SEM). Images were produced from two periods: 4 and 16 h following incubation of the BMC consortium and the rotifer cultures. Starved rotifers without BMC inoculation were used as a control treatment, and performed in parallel with the rotifer-BMC interaction treatments. BEEM-modified capsules (pre-shaped polyethylene molds with hinged lids) with a 100-μm polyester mesh filter were used to initiate the sample treatment procedure for SEM. The rotifers were fixed in 2.5% glutaraldehyde and 0.1 M sodium cacodylate buffer for 1 h and then washed three times with 0.1 M sodium cacodylate buffer solution and sterile seawater. Samples were post-fixed using 1% osmium tetroxide for 1 h at room temperature and washed three times with 0.1 M sodium cacodylate buffer solution and sterile seawater. A series of dehydration washes were performed in ethanol (30, 50, 70, 90, and 100% concentration) for 10 min each, with the final 100% ethanol step repeated three times. Samples were CO_2_ critical-point dried, metallized with gold, and observed in an EVO MA10 scanning electron microscope (Zeiss, Germany) with a voltage between 1 and 30 kV.

### Coral Uptake of BMC-Enriched Rotifers

To monitor uptake of rotifers by corals, one nubbin of *P. damicornis* was transferred to a 1-L aquarium containing 0.22 μm filtered natural seawater at 25°C, salinity of 3.2%, and a magnetic stirrer to circulate the water. To normalize the concentration of rotifers/mL, the culture was filtered using 100 μM, and reinoculated in 50mL of 0.22 μm filtered natural seawater. The concentration of rotifers was calculated by counting the number of rotifers in 3 × 1 mL replicates of water samples from the previously concentrated samples, using a stereomicroscope (Digilab Zoom binocular stereomicroscope, Brazil). Appropriate dilutions were performed to achieve the desired final concentration of 200 rotifers mL^–1^. Before added to the coral aquarium, 100 mL of the culture, containing ∼20,000 rotifers, was washed twice in 0.22-μm filtered seawater to clean the maximum of autofluorescence particles that could interfere with the results. Also, the 10-cm coral nubbin acclimatized in the aquarium for 20 min before the rotifers were inoculated. Video and still images to search for *in vivo* evidence of rotifer uptake by the corals were taken with a USB digital microscope (Digital USB Microscope, Alloet, China).

## Results

**FIGURE 2:**
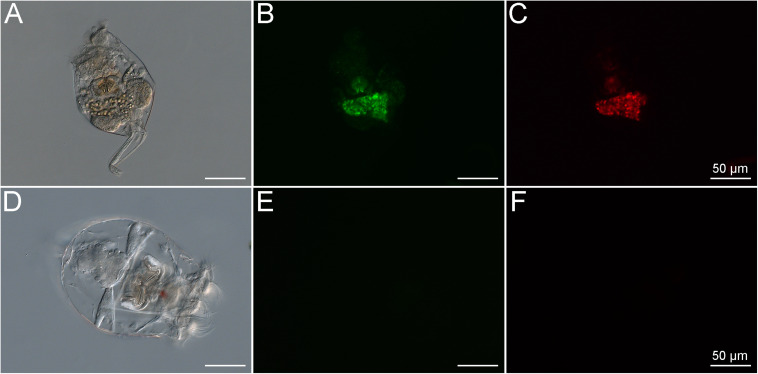
*Brachionus plicatilis* in standard culture conditions observed in differential interference contrast (DIC) **(A)** and fluorescence microscopy, using the Filter set 10 (Zeiss, Germany) **(B)** and the Filter set 00 (Zeiss, Germany) **(C)**. Note that the internal structures of the rotifer show high autofluorescence signals at the same wavelength as both stains used in the LIVE/DEAD^TM^ BacLight^TM^ Bacterial Viability Kit. This shows that the rotifers take up microbes containing pigments with autofluorescence, probably photosynthetic microorganisms. *B. plicatilis* cultured after fasting, observed in differential interference contrast (DIC) **(D)** and fluorescence microscopy using the GFP filter **(E)** and the Rhodamine filter **(F)**. Note the absence of autofluorescence after starvation. This shows that the starvation period was crucial for the success of the experiment.

*Brachionus plicatilis* rotifers cultured under standard conditions (natural unfiltered seawater, at 25°C, salinity of 3.2%) and fed the alga *Tetraselmis gracilis* displayed autofluorescence signals due to uptake of the algae, which contain photosynthetic pigments that are naturally fluorescent (Figures 2A–C). After 7 days of starvation in 0.22-μm filtered seawater, the rotifers displayed no signs of autofluorescence as confirmed by epifluorescence microscopy (Figures 2D–F).

**FIGURE 3:**
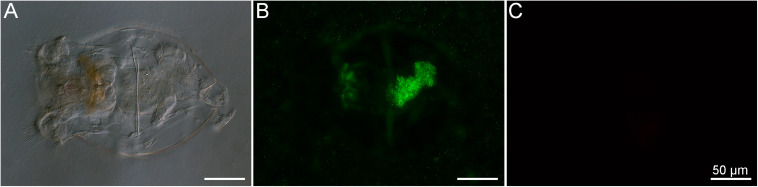
*Brachionus plicatilis* after incubation with the BMC consortium previously stained with the LIVE/DEAD^TM^ BacLight^TM^ Bacterial Viability Kit observed in differential interference contrast (DIC) **(A)** and fluorescence microscopy, using the Filter set 10 (Zeiss, Germany) **(B)** and the Filter set 00 (Zeiss, Germany) **(C)**. Using this kit, viable bacteria cells stain in green while dead cells stain in red. Note that the rotifer ingested a large number of viable bacterial cells (stained in green), resulting in accumulation of stained cells in the stomach and intestine. Bacterial cells also accumulated on the surface of the rotifer, suggesting another possible means of transport of BMC cells.

The LIVE/DEAD bacterial assay was used to demonstrate that the members of the BMC consortium, containing five strains of *Pseudoalteromonas* spp., one of *C. marina*, and one of *H. taeanensis* (Supplementary Table 1), were viable prior to addition to the rotifer cultures (Figure 3). Using the same staining assay, the uptake of fluorescently stained BMCs by starved rotifers was observed, using fluorescence microscopy. Large numbers of bacterial cells were ingested within a few minutes after the BMC consortium was inoculated into the rotifer culture (Supplementary Video 1). In parallel, we were able to follow the pathway of BMCs into the rotifer digestive system *in vivo*, by observing the accumulation of fluorescence in their digestive tract. In addition, we observed strong fluorescence on the external surface of the rotifer body, likely due to the presence of stained BMCs on these surfaces (Figure 3).

**FIGURE 4:**
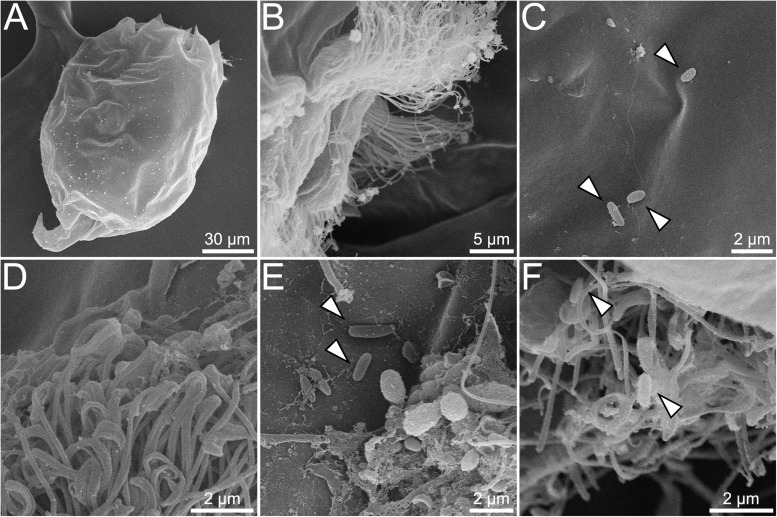
SEM showing interaction between rotifer *Brachionus plicatilis* and BMCs. Control, with no bacteria visible on the surface of *B. plicatilis* **(A)**. Higher magnification of control rotifer, showing no bacteria adhered to the corona cilia **(B)**. Rotifer interaction with BMCs after 4 h **(C,D)**. Higher magnification shows BMCs with rod-shaped bacteria adhered to the rotifer surface **(C)** (white arrowheads). Corona cilia, showing no BMCs adhered to the rotifer corona cilia **(D)**. Rotifer interaction with BMCs after 16 h, showing rod-shaped bacteria adhered to the rotifer surface **(E)** and corona cilia **(F)** (white arrowheads).

Controls (starved, non-BMC-inoculated rotifers) can be visualized with under SEM and show no bacteria interacting with rotifers (Figures 4A,B). Contrarily, SEM revealed the presence of bacteria attached to the corona cilia and rotifer surface after 4 h of interaction (Figures 4C,D); after 16 h, bacteria were also observed on the body surface (Figure 4E) and corona cilia (Figure 4F).

**FIGURE 5:**
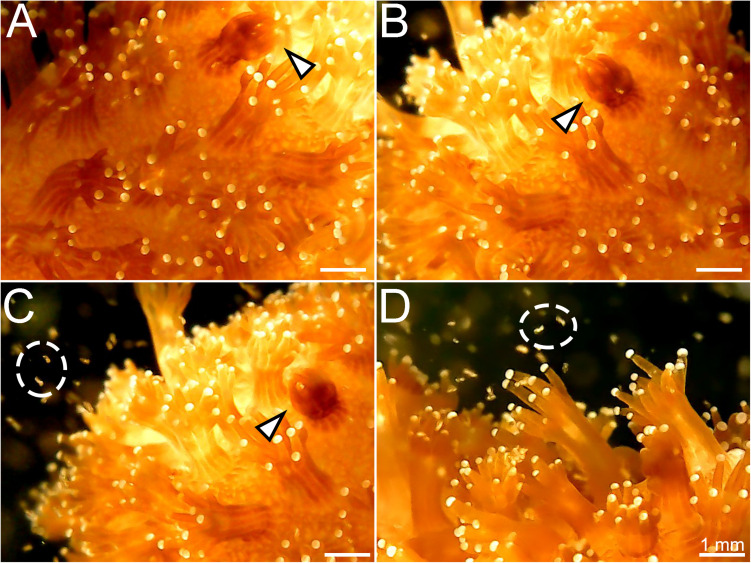
Stages of capture of the rotifer *Brachionus plicatilis* by the coral *Pocillopora damicornis*. **(A)** Coral polyp starting to contract after capturing the rotifer (white arrowhead); **(B)** Polyp totally contracted, engulfing the rotifer; **(C)** The contracted polyp (white arrowhead) and rotifers swimming around the colony (white circle); **(D)** Coral polyps widely extended to capture the rotifers (white circle).

Finally, rotifers fed with BMCs were added to an aquarium containing nubbins of *P. damicornis*. Using the digital microscope, polyps were seen capturing and ingesting the rotifers containing BMCs (Figure 5 and Supplementary Video 2).

## Discussion

Rotifers are commonly used in aquaculture settings to feed fish and shrimp larvae because they are easy to grow, and, through diet supplementation, they can deliver essential nutrients (Lubzens et al., 1989). *Brachionus plicatilis* has been widely used for these purposes because of its indiscriminate feeding behavior (Watanabe et al., 1983; Costa et al., 2016) and tolerance to a wide range of salinity levels, making it an ideal organism for transitioning between culture settings and field applications (Lowe et al., 2005).

In this study, we aimed to alter the internal microbiome of *B. plicatilis* to use this species of rotifer as a possible vector for delivery of specific beneficial bacteria to corals. Previous studies have shown that rotifers are hosts to several symbiotic bacteria, but gnotobiotic rotifers are relatively easy to obtain by changing the composition of their diet (Tinh et al., 2006; Qi et al., 2009). Additionally, it has been shown that *Lactobacillus* probiotic treatment, delivered by rotifers, positively affected growth, survival, and resistance of western white shrimp in aquaculture (Najmi et al., 2018). Here, we show that the BMC strains isolated from corals seemed to accumulate inside and outside the rotifers body in a stable way, suggesting that rotifers are also a promising vector to deliver probiotics for corals. However, further studies need to be performed to elucidate the viability of the bacteria delivered by the rotifers in the coral body. By optimizing this rotifers-based delivery system and selecting BMC strains with potential to face specific threats, we hope to be able to increase coral resistance against adverse stress conditions, such as ocean warming, diseases, and toxic compounds.

A high proportion of the inoculated BMC consortium likely remained viable throughout the duration of the experiments. The BMC consortium was live/dead-stained prior to inoculation into the rotifer culture, to avoid staining non-BMC bacteria associated with the native rotifers. This confirmed the viability of the BMC bacteria prior to inoculation, although we were unable to determine the viability of BMCs within the rotifers after the uptake. The ingestion rate of the rotifers is indirectly related to digestion and nutrient assimilation rates (Salt, 1987). The 7-day starvation period followed by the high availability of BMC cells potentially increased the rate of bacterial ingestion by the rotifers, and, in parallel, decreased the digestion and assimilation rates (Salt, 1987), thereby prolonging the survival of ingested BMCs.

The bacteria selected to be part of the BMC consortium consisted of five strains of *Pseudoalteromonas* spp., one of *C. marina*, and one of *H. taeanensis*, that, as a consortium, have been used to mitigate the harmful effects of the coral pathogen *Vibrio coralliilyticus* in a high-temperature stress scenario (Rosado et al., 2019). Strains of *Pseudoalteromonas* species have been commonly observed adhering to the surfaces of eukaryotic cells (Holmström and Kjelleberg, 1999; Thomas et al., 2008; Goulden et al., 2013). For example, a probiotic strain of *Pseudoalteromonas* selectively attached to external surfaces of both the vector organism *Artemia* and lobster larvae, when introduced as part of a probiotic mixture (Goulden et al., 2013). Here, a strong fluorescent signal consistent with stained bacteria was observed on the external surfaces of the rotifers, and these were potentially some of the *Pseudoalteromonas* spp. strains present in the BMC consortium (Figure 3). Many *Pseudoalteromonas* strains produce antifouling compounds that can prevent the growth or adhesion of other microorganisms (Holmström and Kjelleberg, 1999; Holmström et al., 2002), including a class of bacteriostatic and amphiphilic anti-*Vibrio* molecules (Aranda et al., 2012). The *Pseudoalteromonas* spp. strains included in the BMC consortium may therefore be able to suppress unwanted surface attachment to BMC-enriched rotifers by other opportunistic bacteria before the rotifers are ingested by corals. *Pseudoalteromonas* spp. strains also protect the gastric region in corals by killing *Vibrio* pathogens at high temperatures (Tang et al., 2019). All BMC strains used in this study also possess high catalase activity, which can reduce damage from reactive oxygen species (ROS) that are produced in response to stress on the coral (Peixoto et al., 2017; Rosado et al., 2019). Six of the strains (five *Pseudoalteromonas* spp. and one *Halomonas taeanensis*) contain enzymes involved in nitrogen fixation or sulfur cycling (Rosado et al., 2019), metabolic processes that may underpin nutrient cycling within the coral holobiont (Raina et al., 2009; Rädecker et al., 2015).

Beneficial Microorganisms for Coral studies are targeted toward delivering microbial communities with traits that promote coral health. Delivery of these BMCs through prey items such as rotifers has proven to be possible, with *P. damicornis* corals readily ingesting the rotifers, representing a promising directed-delivery system for bacteria that provide putative beneficial functions for corals. The rotifer itself also provides a nutritional benefit to stressed corals. For example, *Acropora cervicornis* increased its feeding rate of the rotifer *B. plicatilis* under high CO_2_ conditions, and this raised the coral’s total lipid content, which can be a proxy for coral health (Towle et al., 2015). The next steps in establishing this proof of concept consist of determining whether the BMCs delivered through prey items such as rotifers can establish a symbiosis with the coral and provide benefits to the host, and the duration of these associations and benefits.

In summary, this study demonstrated that BMC-enriched rotifers can serve as direct vectors for delivering BMCs to the coral *P. damicornis*. The rotifers freely ingested the BMC consortium, which accumulated in the digestive tract and on the surface. The rotifers were also captured and ingested by the coral polyps, demonstrating the efficacy of using rotifers as a BMC delivery system. These observations represent a step forward in the administration of coral probiotics, which has the potential to increase the persistence and resistance of corals. These findings are also important for probiotic delivery to a wide range of aquaculture species for which rotifers are used as live feed. Further studies to test this approach in the field and to determine whether BMCs delivered via rotifers are able to establish within the coral microbiome will advance our knowledge of the proper administration of beneficial microorganisms *in situ*.

## Data Availability Statement

The original contributions presented in the study are included in the article/Supplementary Material, further inquiries can be directed to the corresponding author/s.

## Author Contributions

RP, DB, LH, FA, and JMA conceived and designed the study. JMA, RFV, GD, and RV maintained the coral and rotifer cultures. JMA, IT, and FA performed the microscopy experiments. RP, JMA, FA, HV, and AB drafted the manuscript. RP provided financial support. All authors were involved in critical revision.

## Conflict of Interest

The authors declare that the research was conducted in the absence of any commercial or financial relationships that could be construed as a potential conflict of interest.

## Supplementary Material

Supplementary Figure 1 Differential interference contrast (DIC) of the rotifer Brachionus plicatilis. The image was taken with a Zeiss Axio Imager D2 microscope (Carl Zeiss, Oberkochen, Germany). Visible morphological features include the corona cilia (purple arrowhead), mouth (blue arrowhead), eye (red arrowhead), mastax (yellow arrowhead), intestine (green arrowhead) and anus (orange arrowhead).

Supplementary Table 1 BMC strains isolated by Rosado et al. (2019) used in this experiment and their respective accession numbers.

Supplementary Video 1 *In vivo* fluorescence microscopy showing the fluorescently stained BMCs being ingested by the starved rotifer within 10 minutes of the interaction. The video was recorded with a Zeiss Axio Imager D2 microscope (Carl Zeiss, Oberkochen, Germany) fitted with a GFP filter. (GFP filter details: Zeiss filter set 09 - Excitation: BP 332 450–490 nm; Beam splitter: FT 510 nm; Emission: LP 515 nm).

Supplementary Video 2 Pocillopora damicornis polyps capturing *Brachionus plicatilis* rotifers. The video was recorded with a digital USB microscope (Alloet, China).

## References

[B1] Aranda C. P. Valenzuela C. Barrientos J. Paredes J. Leal P. Maldonado M. (2012). Bacteriostatic anti-*Vibrio Parahaemolyticus* activity of *Pseudoalteromonas* sp. strains DIT09, DIT44 and DIT46 isolated from Southern Chilean intertidal *Perumytilus purpuratus*. *World J. Microbiol. Biotechnol.* 28 2365–2374. 10.1007/s11274-012-1044-z 22806110

[B2] Bourne D. G. Munn C. B. (2005). Diversity of bacteria associated with the coral *Pocillopora damicornis* from the Great Barrier Reef. *Environ. Microbiol.* 7 1162–1174. 10.1111/j.1462-2920.2005.00793.x 16011753

[B3] Casas I. A. Dobrogosz W. J. (2000). Validation of the probiotic concept: *Lactobacillus reuteri* confers broad-spectrum protection against disease in humans and animals. *Microb. Ecol. Health Dis.* 12 247–285. 10.1080/08910600050216246-1

[B4] Costa A. P. L. Calado R. Marques B. Lillebø A. I. Serôdio J. Soares A. M. V. M. (2016). The effect of mixotrophy in the ex situ culture of the soft coral *Sarcophyton* cf. glaucum. *Aquaculture* 452 151–159. 10.1016/j.aquaculture.2015.10.032

[B5] Damjanovic K. van Oppen M. J. H. Menéndez P. Blackall L. L. (2019). Experimental inoculation of coral recruits with marine bacteria indicates scope for microbiome manipulation in *Acropora tenuis* and *Platygyra daedalea*. *Front. Microbiol.* 10:1702. 10.3389/fmicb.2019.01702 31396197 PMC6668565

[B6] Daniels C. L. Merrifield D. L. Ringø E. Davies S. J. (2013). Probiotic, prebiotic and synbiotic applications for the improvement of larval European lobster (*Homarus gammarus*) culture. *Aquaculture* 416 396–406. 10.1016/j.aquaculture.2013.08.001

[B7] Duarte G. A. S. Villela H. D. M. Deocleciano M. Silva D. Barno A. Cardoso P. M. (2020). Heat waves are a major threat to turbid coral reefs in Brazil. *Front. Mar. Sci.* 7:179 10.3389/fmars.2020.00179

[B8] Dunne C. Murphy L. Flynn S. O’Mahony L. O’Halloran S. Feeney M. (1999). Probiotics: from myth to reality. Demonstration of functionality in animal models of disease and in human clinical trials. Antonie van Leeuwenhoek. *Int. J. Gen. Mol. Microbiol.* 76 279–292. 10.1007/978-94-017-2027-4_14 10532384

[B9] Goulden E. F. Hall M. R. Pereg L. L. Baillie B. K. Høj L. (2013). Probiont niche specialization contributes to additive protection against *Vibrio owensii* spiny lobster larvae. *Environ. Microbiol. Rep.* 5 39–48. 10.1111/1758-2229.12007 23757129

[B10] Hai N. V. (2015). The use of probiotics in aquaculture. *J. Appl. Microbiol.* 119 917–935. 26119489 10.1111/jam.12886

[B11] Hai N. V. Buller N. Fotedar R. (2010). Encapsulation capacity of *Artemia* nauplii with customized probiotics for use in the cultivation of western king prawns (*Penaeus latisulcatus* Kishinouye, 1896). *Aquac. Res.* 41 893–903. 10.1111/j.1365-2109.2009.02370.x

[B12] Holmström C. Kjelleberg S. (1999). Marine *Pseudoalteromonas* species are associated with higher organisms and produce biologically active extracellular agents. *FEMS Microbiol. Ecol.* 30 285–293. 10.1016/s0168-6496(99)00063-x 10568837

[B13] Holmström C. Egan S. Franks A. McCloy S. Kjelleberg S. (2002). Antifouling activities expressed by marine surface associated *Pseudoalteromonas* species. *FEMS Microbiol. Ecol.* 41 47–58. 10.1016/s0168-6496(02)00239-8 19709238

[B14] Hughes T. P. Anderson K. D. Connolly S. R. Heron S. F. Kerry J. T. Lough J. M. (2018). Spatial and temporal patterns of mass bleaching of corals in the Anthropocene. *Science* 359 80–83. 10.1126/science.aan8048 29302011

[B15] Lowe C. D. Kemp S. J. Bates A. D. Montagnes D. J. S. (2005). Evidence that the rotifer *Brachionus plicatilis* is not an osmoconformer. *Mar. Biol.* 146 923–929. 10.1007/s00227-004-1501-9

[B16] Lubzens E. Tandler A. Minkoff G. (1989). Rotifers as food in aquaculture. *Hydrobiologia* 18 387–400. 10.1007/bf00048937

[B17] Najmi N. Yahyavi M. Haghshena A. (2018). Effect of enriched rotifer (*Brachionus plicstilis*) with probiotic lactobacilli on growth, survival and resistance indicators of western white shrimp (*Litopenaeus vannamei*) larvae. *Iran. J. Fish. Sci.* 17 11–20.

[B18] National Academies of Sciences and Medicine (2019). *A Research Review of Interventions to Increase the Persistence and Resilience of Coral Reefs. Consensus Study Report.* Washington, DC: The National Academies Press, 10.17226/25279

[B19] Pandolfi J. M. Connolly S. R. Marshall D. J. Cohen A. L. (2011). Projecting coral reef futures under global warming and ocean acidification. *Science* 333 418–422. 10.1126/science.1204794 21778392

[B20] Peixoto R. Rosado P. M. Leite D. C. de A. Rosado A. S. Bourne D. G. (2017). Beneficial microorganisms for corals (BMC): proposed mechanisms for coral health and resilience. *Front. Microbiol.* 8:341. 10.3389/fmicb.2017.00341 28326066 PMC5339234

[B21] Pita L. Rix L. Slaby B. M. Franke A. Hentschel U. (2018). The sponge holobiont in a changing ocean: from microbes to ecosystems. *Microbiome* 6:46. 10.1186/s40168-018-0428-1 PMC5845141 29523192

[B22] Planas M. Pérez-Lorenzo M. Hjelm M. Gram L. Fiksdal I. U. Bergh Ø, et al. (2006). Probiotic effect in vivo of *Roseobacter* strain 27-4 against *Vibrio* (*Listonella*) *anguillarum* infections in turbot (*Scophthalmus maximus* L.) larvae. *Aquaculture* 255 323–333. 10.1016/j.aquaculture.2005.11.039

[B23] Planas M. Pérez-Lorenzo M. Vázquez J. A. Pintado J. (2005). A model for experimental infections with *Vibrio* (*Listonella*) *anguillarum* in first feeding turbot (*Scophthalmus maximus* L.) larvae under hatchery conditions. *Aquaculture* 250 232–243. 10.1016/j.aquaculture.2005.04.050

[B24] Qi Z. Dierckens K. Defoirdt T. Sorgeloos P. Boon N. Bao Z. (2009). Effects of feeding regime and probionts on the diverting microbial communities in rotifer *Brachionus* culture. *Aquac. Int.* 17 303–315. 10.1007/s10499-008-9202-x

[B25] Rädecker N. Pogoreutz C. Voolstra C. R. Wiedenmann J. Wild C. (2015). Nitrogen cycling in corals: the key to understanding holobiont functioning? *Trends Microbiol*. 23 490–497. 10.1016/j.tim.2015.03.008 25868684

[B26] Raina J.-B. Tapiolas D. Willis B. L. Bourne D. G. (2009). Coral-associated bacteria and their role in the biogeochemical cycling of sulfur. *Appl. Environ. Microbiol.* 75 3492–3501. 10.1128/aem.02567-08 19346350 PMC2687302

[B27] Rohwer F. Breitbart M. Jara J. Azam F. Knowlton N. (2001). Diversity of bacteria associated with the Caribbean coral *Montastraea franksi*. *Coral Reefs* 20 85–91. 10.1007/s003380100138

[B28] Roobab U. Batool Z. Manzoor M. F. Shabbir M. A. Khan M. R. Aadil R. M. (2020). Sources, formulations, advanced delivery and health benefits of probiotics. *Curr. Opin. Food Sci.* 32 17–28. 10.1016/j.cofs.2020.01.003

[B29] Rosado P. Leite D. C. A. Duarte G. A. S. Chaloub R. M. Jospin G. Nunes da Rocha U. (2019). Marine probiotics: increasing coral resistance to bleaching through microbiome manipulation. *ISME J.* 13 921–936. 10.1038/s41396-018-0323-6 30518818 PMC6461899

[B30] Salt G. W. (1987). The components of feeding behavior in rotifers. *Hydrobiologia* 147 271–281. 10.1007/bf00025754

[B31] Sánchez-Quinto A. Falcón L. I. (2019). Metagenome of *Acropora palmata* coral rubble: potential metabolic pathways and diversity in the reef ecosystem. *PLoS One* 14:e0220117. 10.1371/journal.pone.0220117 31394568 PMC6687439

[B32] Santos H. F. A. Duarte G. A. S. Rachid C. T. Chaloub R. M. Calderon E. N. Marangoni L. F. (2015). Impact of oil spills on coral reefs can be reduced by bioremediation using probiotic microbiota. *Sci. Rep.* 5:18268. 10.1038/srep18268 PMC4677405 26658023

[B33] Stolzenbach S. Myhill L. J. Andersen L. O. Krych L. Mejer H. Williams A. R. (2020). Dietary inulin and *Trichuris suis* infection promote beneficial bacteria throughout the porcine gut. *Front. Microbiol.* 11:312. 10.3389/fmicb.2020.00312 32194529 PMC7064446

[B34] Sun Y-Z. Yang H-L. Huang K-P. Ye J-D. Zhang C-X. (2013). Application of autochthonous Bacillus bioencapsulated in copepod to grouper *Epinephelus coioides* larvae. *Aquaculture* 392:44–50. 10.1016/j.aquaculture.2013.01.037

[B35] Tang K. Zhan W. Zhou Y. Xu T. Chen X. Wang W. (2019). Antagonism between coral pathogen *Vibrio coralliilyticus* and other bacteria in the gastric cavity of scleractinian coral *Galaxea fascicularis*. *Sci. China Earth Sci.* 63 157–166. 10.1007/s11430-019-9388-3

[B36] Teplitski M. Ritchie K. (2009). How feasible is the biological control of coral diseases? *Trends Ecol. Evol.* 24 378–385. 10.1016/j.tree.2009.02.008 19406502

[B37] Thomas T. Evans F. F. Schleheck D. Mai-Prochnow A. Burke C. Penesyan A. (2008). Analysis of the Pseudoalteromonas tunicata genome reveals properties of a surface-associated life style in the marine environment. *PLoS One* 3:e3252. 10.1371/journal.pone.0003252 18813346 PMC2536512

[B38] Tinh N. T. N. Phuoc N. N. Dierckens K. Sorgeloos P. Bossier P. (2006). Gnotobiotically grown rotifer *Brachionus plicatilis* sensu strictu as a tool for evaluation of microbial functions and nutritional value of different food types. *Aquaculture* 253 421–432. 10.1016/j.aquaculture.2005.09.006

[B39] Towle E. K. Enochs I. C. Langdon C. (2015). Threatened Caribbean coral is able to mitigate the adverse effects of ocean acidification on calcification by increasing feeding rate. *PLoS One* 10:e0123394. 10.1371/journal.pone.0123394 25874963 PMC4398515

[B40] Watanabe T. Kitajima C. Fujita S. (1983). Nutritional values of live organisms used in Japan for mass propagation of fish: a review. *Aquaculture* 34 115–143. 10.1016/0044-8486(83)90296-x

[B41] Wilkins L. G. E. Leray M. O’Dea A. Yuen B. Peixoto R. S. Pereira T. J. (2019). Host-associated microbiomes drive structure and function of marine ecosystems. *PLoS Biol.* 17:e3000533. 10.1371/journal.pbio.3000533 31710600 PMC6874084

