# Supplementary figures and images for: Delivering Beneficial Microorganisms for Corals: Rotifers as Carriers of Probiotic Bacteria
Source: Front Microbiol. 2020 Dec 15;11:608506. doi: 10.3389/fmicb.2020.608506 (PMC7769773; doi:10.3389/fmicb.2020.608506)

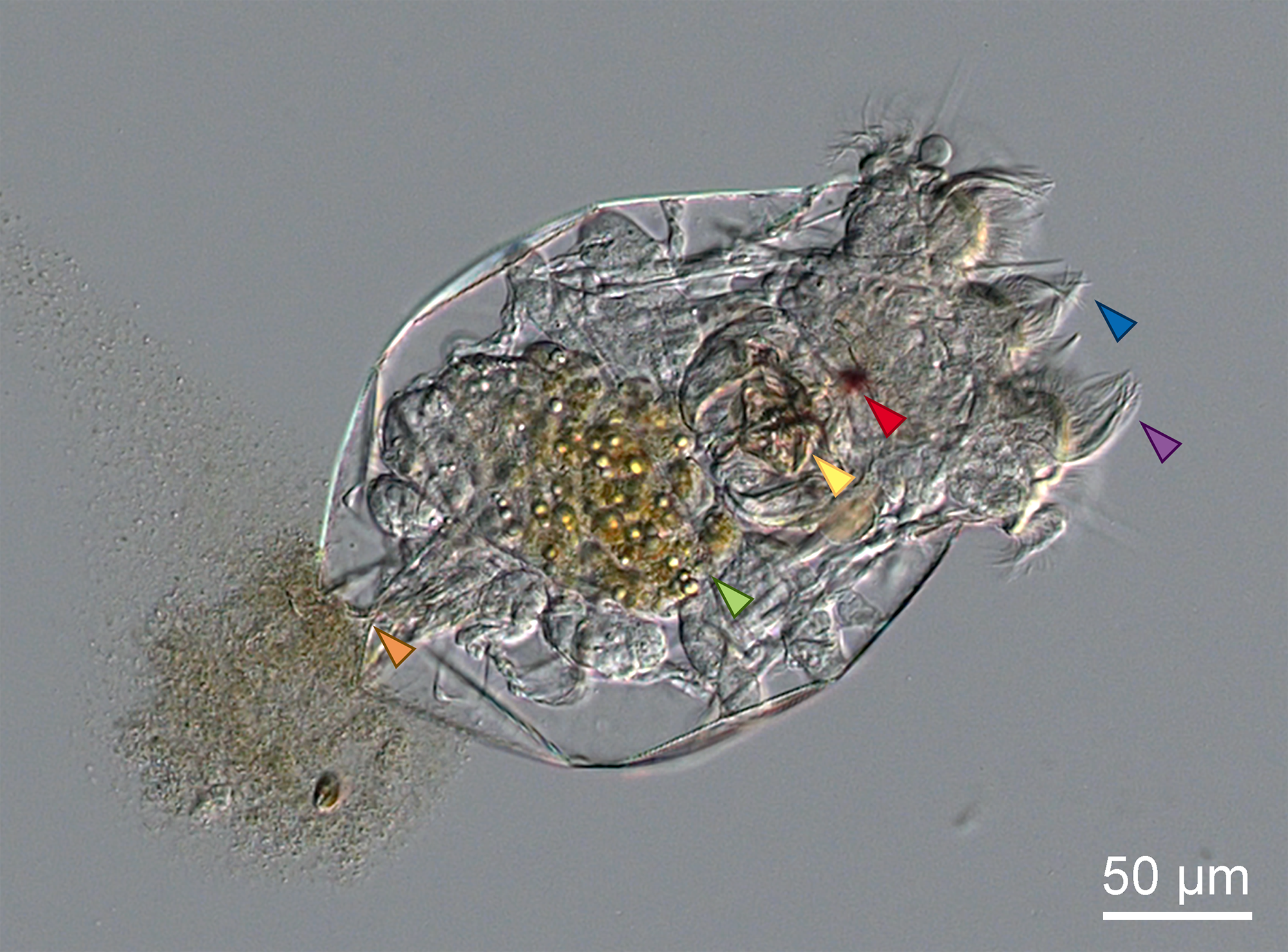

Supplement: Supplementary Figure 1 — Differential interference contrast (DIC) of the rotifer Brachionus plicatilis. The image was taken with a Zeiss Axio Imager D2 microscope (Carl Zeiss, Oberkochen, Germany). Visible morphological features include the corona cilia (purple arrowhead), mouth (blue arrowhead), eye (red arrowhead), mastax (yellow arrowhead), intestine (green arrowhead) and anus (orange arrowhead). [file Image_1.TIF]
